# Abnormal tracheal smooth muscle function in the CF mouse

**DOI:** 10.1002/phy2.138

**Published:** 2013-11-05

**Authors:** Helen L Wallace, Kevin W Southern, Marilyn G Connell, Susan Wray, Theodor Burdyga

**Affiliations:** 1 Department of Cellular and Molecular Physiology, Institute of Translational Medicine, University of LiverpoolLiverpool, U.K.; 2 Department for Women's and Children's Health, Institute of Translational Medicine, University of LiverpoolLiverpool, U.K.

**Keywords:** Contraction, cystic fibrosis, trachea, smooth muscle

## Abstract

Increased airway smooth muscle (ASM) contractility is thought to underlie symptoms of airway hyperresponsiveness (AHR). In the cystic fibrosis (CF) airway, ASM anomalies have been reported, but have not been fully characterized and the underlying mechanisms are largely unknown. We examined ASM in an adult CF mouse tracheal ring preparation, and determined whether changes in contractility were associated with altered ASM morphology. We looked for inherent changes in the cellular pathways involved in contractility, and characterized trachea morphology in the adult trachea and in an embryonic lung culture model during development. Results showed that that there was a reduction in tracheal caliber in CF mice as indicated by a reduction in the number of cartilage rings; proximal cross-sectional areas of *cftr*^*−/−*^ tracheas and luminal areas were significantly smaller, but there was no difference in the area or distribution of smooth muscle. Morphological differences observed in adult trachea were not evident in the embryonic lung at 11.5 days gestation or after 72 h in culture. Functional data showed a significant reduction in the amplitude and duration of contraction in response to carbachol (CCh) in Ca-free conditions. The reduction in contraction was agonist specific, and occurred throughout the length of the trachea. These data show that there is a loss in the contractile capacity of the CF mouse trachea due to downregulation of the pathway specific to acetylcholine (ACh) activation. This reduction in contraction is not associated with changes in the area or distribution of ASM.

## Introduction

Excessive contraction of airway smooth muscle (ASM) is thought to be responsible for airway hyperresponsiveness (AHR), a predominant feature of asthma. Hyperresponsive airways show increased responsiveness to certain bronchoconstrictors, and the resulting airway obstruction is acutely responsive to bronchodilators. Fundamental mechanisms underlying increased ASM contractility and airway narrowing are still largely unknown (Govindaraju et al. 2008). Patients with cystic fibrosis (CF) may also suffer from asthma-type symptoms, although diagnosis is often difficult due to the underlying CF condition. Early reports of AHR in CF were shown to be greater in CF patients with more severe lung disease, occurring secondary to bronchial damage. It was therefore suggested that underlying airway narrowing and thickening may contribute to AHR in CF (Weinberger 2002). However, the lack of evidence for the effectiveness of bronchodilators (Halfhide et al. 2005) in the CF airway questions the role of increased ASM contraction in CF disease.

The consequences of defective cystic fibrosis transmembrane conductance regulator (CFTR) on epithelial cell function have been widely studied in the CF airway, but the effect on other cells is not as well documented. CFTR is expressed in ASM cells (Vandebrouck et al. 2006) suggesting a role for CFTR in ASM function. An increase in ASM content has been described in the lower airway of adults and children with CF suggesting structural changes to ASM occur early in the development of CF airway disease (Regamey et al. 2008). A study on ASM cells of CF patients suggests that CF ASM cells are more contractile in response to the cytokine IL-8 which is known to be elevated in CF (Govindaraju et al. 2008; Tabary et al. 1998). Another study on rat tracheal ASM suggests that CFTR is important for bronchodilation, implying that lack of CFTR may result in an increase in bronchoconstriction (Vandebrouck et al. 2006). Other studies have pointed toward a reduction in ASM function; one study on human cultured ASM cells reported a reduction in Ca release in response to histamine stimulation in CF cells, suggesting that CFTR regulates release of Ca in response to contractile agents (Michoud et al. 2009). Another recent study showed a decrease in the sensitivity of the contractile response of tracheal smooth muscle to the agonist carbachol (CCh) in transgenic CF mice (Bonvin et al. 2008). This decrease was reported to be due to observed abnormalities in cartilage ring formation and a loss of structural support toward the proximal end of the trachea. Sensitivity to CCh was reported to be normal at the distal end of the trachea where structural anomalies were less evident. Thus, while there are suggestions of reduced sensitivity to CCh that is anatomically linked, changes in contractility were not fully characterized and agonists other than CCh were not tested. It is also not known whether changes in ASM function are specifically related to alterations in ASM morphology. The question of how early in the disease process tracheal changes occur remains to be determined. During development, lung growth is thought to be dependent on ASM peristalsis which can initiate from the trachea (Jesudason et al. 2005). We have previously shown that lung growth and peristalsis do not appear abnormal in CF mice (Wallace et al. 2008); however, it is not known whether structural abnormalities and a reduction in trachea caliber are evident at this stage in development.

In this study, we aimed to elucidate the ASM abnormalities in the CF trachea of transgenic CF mice. We looked for evidence of an association between altered ASM morphology and abnormal ASM function.

We investigated changes in alternate pathways underlying ASM function by measuring contractility in a tracheal ring preparation in response to different stimuli. High-K^+^ depolarization was used to study contractions induced by Ca entry through voltage-gated L-type Ca channels; Histamine, 5-hydroxytryptamine (5-HT), and CCh applied in Ca-free solution were used to characterize contractility associated with Ca release from the sarcoplasmic reticulum (SR); Caffeine was used to test the presence and functional role of ryanodine receptor (RyRs) channels (Burdyga et al. 1995). We also looked for changes in Ca sensitivity of the contractile machinery and the possible involvement of Rho-associated kinase (ROK) in the CF and non-CF airway using the selective ROK inhibitor H-1152(Shabir et al. 2004). Finally, we determined whether structural tracheal changes occur at the early stages of development in an embryonic lung culture model.

## Methods

### Tissue

Heterozygous (^+/*−*^) and homozygous (^*−*/*−*^) *cftr*^*tm1Cam*^ MF1/129 mice were obtained from a colony maintained at our laboratory. The genotype of each individual animal was determined by polymerase chain reaction (PCR) amplification using DNA isolated from ear clippings (DNA extraction using DNeasy tissue kit, Qiagen, Manchester, UK). Tracheas were removed from 6-week-old heterozygous *cftr*^*tm1Cam*^ MF1/129 mice following humane killing in accordance with U.K. legislation, and placed in physiological saline solution. Individual tracheal rings were then dissected and cut into strips of smooth muscle with cartilage remaining at each end.

### Solutions

Tissue was placed in HEPES buffered modified Krebs solution (pH 7.4 adjusted with NaOH) composed of 120 mmol/L NaCl, 5.9 mmol/L KCl, 1.2 mmol/L MgSO4, 2 mmol/L CaCl2, 8 mmol/L glucose, and 11 mmol/L HEPES (Sigma, St Louis, MO). Solutions with increasing [K^+^] were obtained by replacing Na^+^ by equimolar K^+^. Ca-free solutions contained 2 mmol/L ethylene glycol tetraacetic acid (EGTA).

### Contraction measurements

Tracheal strips were secured with aluminum foil clips (Laser Services, Westford, MA) and mounted on two stainless steel hooks, one of which was fixed, and the other was attached to a force transducer recording amplitude of force in mN. The secured strip was then submerged in solution contained in a well plate heated to 35–37°C. The well plate consisting of four separate wells was mounted on a moveable spring system to enable easy transfer of the tissue between wells. Each strip was passively stretched until a change in baseline force was registered. A response to high-K^+^ depolarization was then recorded followed by a further stretch of 25–30% of the maximal force produced by high-K^+^. In preliminary experiments, cumulative concentration-dependent responses were performed for each agonist in order to determine the concentration producing the maximum response (data not shown). Contractility was then manipulated using 50 μmol/L CCh, 40 μmol/L 5H-T, and 20 mmol/L caffeine. Responses were recorded in Krebs and Ca-free solution; the protocol for Ca-free responses consisted of a 40-sec application of KCl (high-K^+^), 2-min application of Ca-free solution, and 1-min application of agonist. Strips were then placed back into Krebs solution for 5 min before repeating the protocol. Individual strips were used for each agonist, and repeated protocols were measured. Responses were also recorded in the presence of the ROK inhibitor H-1152 (0.2 μmol/L) after incubating smooth muscle strips with H-1152 for 15 min.

### Histology

For morphological analysis, whole tracheas were fixed in 4% paraformaldehyde, embedded in 5% gelatine, and snap frozen as described previously (Wallace et al. 2008). Cryosections of 7 μm were taken from the three most proximal rings, stained with hematoxylin and eosin, and labeled with anti-actin, α-smooth muscle-Cy3 monoclonal antibody 1a4 (sigma). Ten sections from each animal selected at regular intervals were observed and photographed under a fluorescent microscope and used for analysis. Area and distribution of smooth muscle was analyzed using image J software (image J 1.34s public domain downloaded from rsb.info.nih.gov/ij/). Circularity of sections was determined using the formula 4π (area/perimeter^2^) with a value of one depicting a perfect circle.

### Embryonic lung cultures

Mice were sacrificed on day 11.5 gestation (equivalent to 37 days postconceptional age in the human) in accordance with U.K. legislation. Lung primordia were harvested and embryo tissue was retained for genotyping (DNA extraction DNeasy tissue kit, Qiagen and DNA amplification on thermocycler). Lungs were transferred to translucent membranes (Millicell, Millipore Corp, Bedford, MA) and cultured for 78 hours at 37°C in 5% (v/v) CO_2_ as described previously (Jesudason et al. 2000). Lungs were cultured at the air-medium interface and were bathed in serum-free culture medium (DMEM/F12; Gibco, Life Technologies, Paisley, UK), with penicillin 100 IU/mL and Streptomycin 100 μg/mL (GibcoBRL, UK).

Lungs were photographed after 6, 30, 54, and 78 h in culture using a Hamamatsu C7190-10 series dual mode cooled EB-CCD camera (Hamamatsu City, Japan) with a 5 × 0.25 na Zeiss Fluar objective (Zeiss, Jena, Germany). Images were captured and processed using kinetic imaging AQM (Nottingham, UK) software. Trachea outlines were traced and measurements were recorded using image J software. Tracheal length, width immediately above the bronchus (width 1) and below the larynx (width 2), and overall area were calculated following calibration with a graticule.

### Statistical analysis

**Figure 1:**
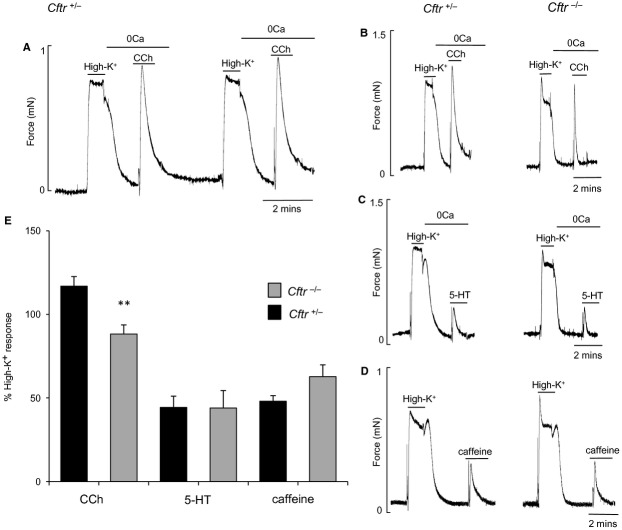
Amplitude of response to high-K^+^, CCh, 5-HT, and caffeine in *cftr*^*+/−*^ and *cftr*^*−/−*^ mice. (A) An individual trace from a *cftr*^*+/−*^ tracheal ring showing consecutive responses to CCh in Ca-free conditions. B–D show comparisons between *cftr*^*+/−*^ and *cftr*^*−/−*^ mice; (B) Responses to CCh in Ca-free conditions, (C) Responses to 5-HT in Ca-free conditions, (D) Responses to caffeine. (E) Mean ± SEM responses to CCh (*cftr*^*−/−*^ *n* = 9, *cftr*^*+/−*^ *n* = 9), 5-HT (*cftr*^*−/−*^ *n* = 5, *cftr*^*+/−*^*n* = 6), and caffeine (*cftr*^*−/−*^ *n* = 5, *cftr*^*+/−*^ *n* = 7). Amplitude of responses is shown as a percentage of high-K^+^ responses. CCh, carbachol. **Denotes significance at 0.01 level.

The mean amplitude (mN) and duration (msec) of contraction were analyzed for each agonist. Amplitude was determined by measuring the peak of contraction; for high-K^+^ responses a peak followed by a lower sustained response was observed (Figs. 1 and 3). In this case, the amplitudes of the peak and sustained components were determined. Amplitude of contraction to CCh, 5H-T, and caffeine were normalized to the peak amplitude of the high-K^+^ response (taken as 100%).

**Figure 2:**
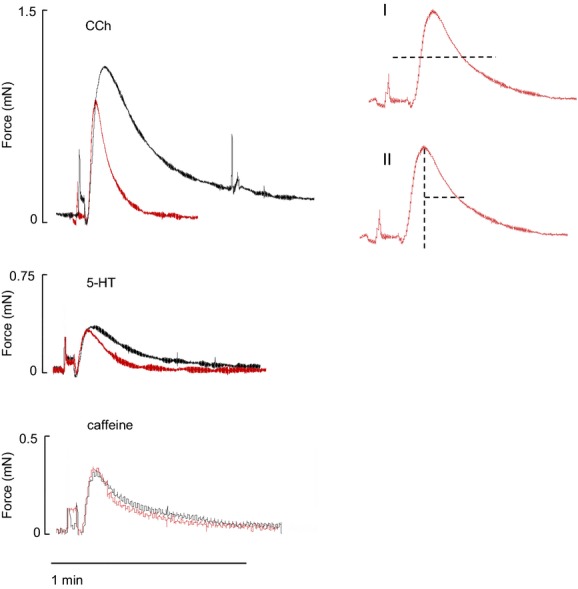
Duration of response to CCh (upper), 5-HT (middle), and caffeine (bottom), *cftr*^*+/−*^ responses are shown in black and *cftr*^*−/−*^ responses in red. Calculation of duration at 50% peak amplitude is shown in I, and the half time of relaxation (time taken to relax to half peak amplitude (*t*_1/2_) is shown in II).

The duration of each contraction was analyzed by determining the duration at 50% peak amplitude, and the half time of relaxation (time taken to relax to half peak amplitude [*t*_1/2_]) as shown in Figure 2. Mean values per strip were determined and then the overall mean for each animal calculated. Responses were recorded in strips taken throughout the length of trachea. Combined data were analyzed, as well as separate analyses of strips divided into proximal and distal strips for comparison. Normality of the data distribution was assessed using the Shapiro–Wilk test; the student's *t-*test or Mann–Whitney *U-*test was applied accordingly, or after H-1152 treatment, the paired Student's *t-*test or Wilcoxon Signed Rank Test for nonparametric data (SPSS Statistics Package version 13 for Windows; SPSS, Chicago, IL). Values are given as means ± SEM and *n* is the number of animals. Significance was taken as *P* < 0.05 throughout.

## Results

### Tracheal smooth muscle contractility in control *cftr*^+/−^ mice

Amplitude of contraction was measured in response to high-K^+^, CCh (50 μmol/L), 5-HT (40 μmol/L), and caffeine (20 mmol/L). Application of high-K^+^ quickly produced a strong contraction that peaked and then reached a lower sustained steady state (sustained component) within 10 sec (Fig. 1A). CCh elicited a similar fast response, in Krebs solution responses reached 182 ± 16.6% of the high-K^+^ response (data not shown). In Ca-free conditions used to assess SR releasable Ca, CCh and 5HT elicited transient contractions (Fig. 1A–D). The amplitude of the CCh-induced contracture (118.5 ± 5.7%), however, was more than two times higher than that induced by either 5-HT (44.3 ± 6.8%) or caffeine (48 ± 3.4%). Histamine failed to produce any contraction as reported previously (Fernandez-Rodriguez et al. 2010).

### Comparison between *cftr*^+/−^ and *cftr*^−/−^ mice

The peak response to high-K^+^ depolarization was not significantly different between groups (0.67 ± 0.08 mN (*cftr*^*+/−*^), *n* = 21; 0.50 ± 0.07 mN (*cftr*^*−/−*^), *n* = 17, *P* = 0.1). The sustained component of the high-K^+^ also showed similar characteristics between groups (79 ± 2.9% of peak high-K^+^ response (*cftr*^*+/−*^), 83 ± 3.2% of peak high-K+ response (*cftr*^*−/−*^), *P* = 0.1). There was no significant difference in response to CCh in Krebs solution between groups when normalized to high-K^+^ (see Methods) (182 ± 16.6% [*cftr*^*+/−*^], 201 ± 12.3% [*cftr*^*−/−*^]). However, when applied in Ca-free conditions, there was a significant decrease in the response of CCh-induced contraction in *cftr*^*−/−*^ tissue compared with controls (89.6 ± 5% [*cftr*^*−/−*^], *n* = 9; 118.5 ± 5% (*cftr*^*+/−*^), *n* = 9, Fig. 1E *P* < 0.01). The duration of CCh-induced contraction measured at 50% of peak in *cftr*^*−/−*^ mice was significantly reduced relative to control (14 ± 2.6 sec [*cftr*^*−/−*^], 30.2 ± 2.4 sec (*cftr*^*+/−*^), *P* < 0.01, Fig. 2). The *t*_1/2_ of relaxation of CCh-induced force transient was also shorter in *cftr*^*−/−*^ mice relative to control (11.2 ± 2.3 sec [*cftr*^*−/−*^], 24.8 ± 2.78 sec [*cftr*^*+/−*^], *P* < 0.01 Mann–Whitney *U-*test). There was no significant difference in the amplitude of response to 5-HT (44.3 ± 6.8% [*cftr*^*+/−*^], *n* = 6; 44 ± 10.5% [*cftr*^*−/−*^], *n* = 5, *P* = 1) or caffeine, (48 ± 3.4% [*cftr*^*+/−*^], *n* = 7; 62.8 ± 7% [*cftr*^*−/−*^], *n* = 5, *P* = 0.1) between groups (Fig. 1). The *t*_1/2_ of relaxation and duration of 5-HT and caffeine- induced contraction were also similar between groups (5-HT *t*_1/2_: 14.1 ± 2.2 [*cftr*^*−/−*^], *n* = 5; 19.1 ± 2.5 [*cftr*^*+/−*^], *n* = 6, *P* = 0.17. Duration: 18.3 ± 1.6 [*cftr*^*−/−*^], *n* = 5; 24.1 ± 2.3 [*cftr*^*+/−*^], *n* = 5, *P* = 0.09. Caffeine *t*_1/2_:9.3 ± 3.4 [*cftr*^*−/−*^], *n* = 5; 9.4 ± 2.5 [*cftr*^*+/−*^] *n* = 5, *P* = 0.17. Duration: 13.5 ± 3.4 [*cftr*^*−/−*^], *n* = 5; 13.3 ± 1.8 [*cftr*^*+/−*^], *n* = 5, *P* = 0.6, Fig. 2). When divided into proximal and distal strips, the reduction in the amplitude of contraction to CCh is shown to occur throughout the trachea (proximal CCh amplitude: 141.8 ± 9.7% [*cftr*^*+/−*^], *n* = 7; 95.7 ± 8.6% [*cftr*^*−/−*^], *n* = 5 *P* < 0.01; distal CCh amplitude: 119.1 ± 13.6% [*cftr*^*+/−*^], *n* = 6; 87.2 ± 4.7% [*cftr*^*−/−*^], *n* = 6, *P* < 0.05).

### Effects of H-1152

**Figure 3:**
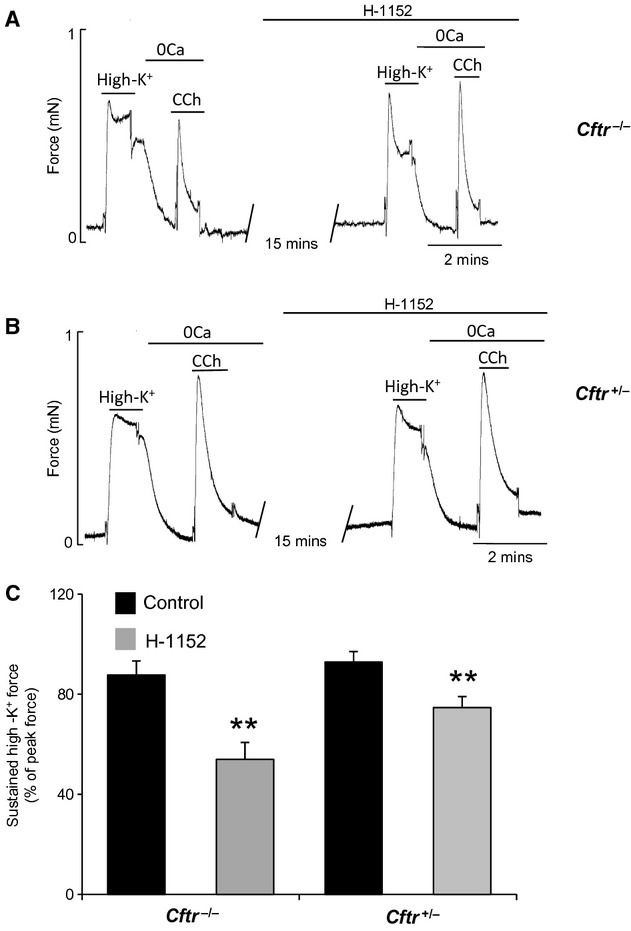
Reduction in the amplitude of the sustained component of the high-K^+^ response after H-1152 application. Traces from (A) *cftr*^*−/−*^, and (B) *cftr*^*+/−*^, before and after 15-min application of H-1152, (C) Reduction in the amplitude of the sustained component of the high-K^+^ response shown as a percentage of the peak high-K^+^ response before (control) and after 15 min application of H-1152 in *cftr*^*−/−*^ and *cftr*^*+/−*^ mice (*n* = 5).

Contractility was further investigated by examining the sensitivity of tracheal ASM to Rho-kinase inhibition. The effect of ROK inhibitor H-1152 on force induced by different stimuli was determined (Fig. 3). After 15 min pretreatment of tracheal smooth muscle with H-1152, there was no change in the CCh response in *cftr*^*+/−*^ mice (Fig. 3B). There appeared to be an increase in response to CCh in *cftr*^*−/−*^ mice after application of H-1152, however, the apparent increase was not significant (0.73 ± 0.19 mN before H1152, 0.85 ± 0.23 mN after H1152, *P* = 0.12, *n* = 5, Fig. 3A). The sustained component of the high-K^+^ response was significantly reduced in both groups, and this inhibitory effect was stronger in *cftr*^*−*/*−*^ mice (18.3% reduction to 74.6 ± 4% of peak high-K^+^ response [*cftr*^*+/−*^], *P* < 0.01; 33.8% reduction to 53.9 ± 6% of peak high-K^+^ response (*cftr*^*−/−*^), *P* < 0.01, Fig. 3C).

These results suggest that ROK plays a more significant role in the maintenance of contraction induced by high-K^+^ depolarization in CF tracheal ASM. Agonist binding has a minimal effect on ROK activation in tracheal smooth muscle of *cftr*^*+/−*^ or *cftr*^*−/−*^ mice.

### Morphology

**Figure 4:**
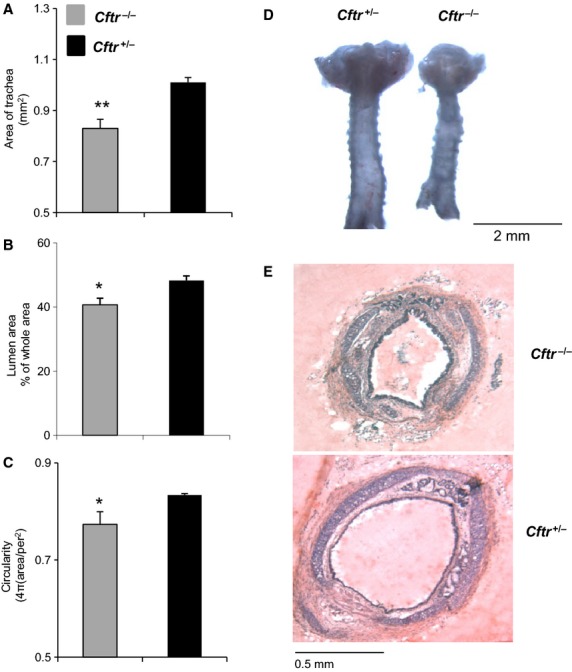
Morphology of tracheal rings in *cftr*^*−/−*^ and *cftr*^*+/−*^ mice. Cumulative data showing (A) Cross-sectional area of trachea, (B) lumen area as a percentage of whole area, and (C) circularity of tracheal lumens. Data represent proximal tracheal ring sections, mean ± SEM, (*n* = 5); ** and *denote significance at 0.01 and 0.05 levels, respectively. (D) Comparison of whole tracheas from *cftr*^*−/−*^ and *cftr*^*+/−*^ mice, and (E) proximal tracheal ring sections stained with hematoxylin and eosin.

**Figure 5:**
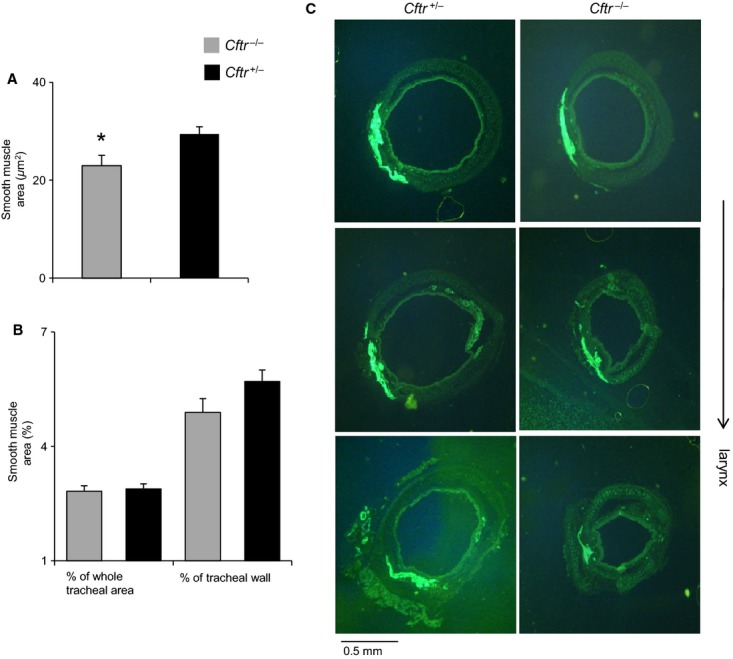
Area and distribution of smooth muscle in tracheal rings of *cftr*^*−/−*^ and *cftr*^*+/−*^ mice. Cumulative data represent proximal tracheal ring sections, mean ± SEM, (*n* = 5); *denotes significance at 0.05 level. (A) Area of smooth muscle. (B) Area of smooth muscle shown as a percentage of whole tracheal area and tracheal wall. (C) Tracheal ring sections viewed under fluorescent microscope highlighting area of smooth muscle fluorescing green. Sections were taken at 200-μm intervals.

The trachea from *cftr*^*−/−*^ mice were consistently smaller with 1–2 fewer cartilage rings observed in 13 of 15 tracheas compared with littermate controls (Fig. 4D, *P* < 0.01). Narrowing at the most proximal part of the trachea was evident in 12 of 15 *cftr*^*−/−*^ tracheas. Proximal cross-sectional areas of trachea were 25.2% smaller (*P* < 0.01) and less circular than controls (0.73 ± 0.02 [*cftr*^*−/−*^], 0.84 ± 0.008 [*cftr*^*+/−*^], *P* < 0.01) (Fig. 4). The luminal area was also significantly smaller when calculated as a percentage of the cross-sectional area (41.3 ± 3.6% [*cftr*^*−/−*^], 49.5 ± 1.6 [*cftr*^*+/−*^]). Sections stained with hematoxylin and eosin indicated the area of epithelium lining the lumen that was increased by 10% in CF tracheas, but these changes were not significant. Sections labeled with anti-actin showed that smooth muscle area was reduced in CF tracheas; however, when normalized to whole trachea area, there was no significant difference between genotypes (2.88 ± 0.18% [*cftr*^*−/−*^]; 3.25 ± 0.3% [*cftr*^*+/−*^] (Fig. 5).

**Figure 6:**
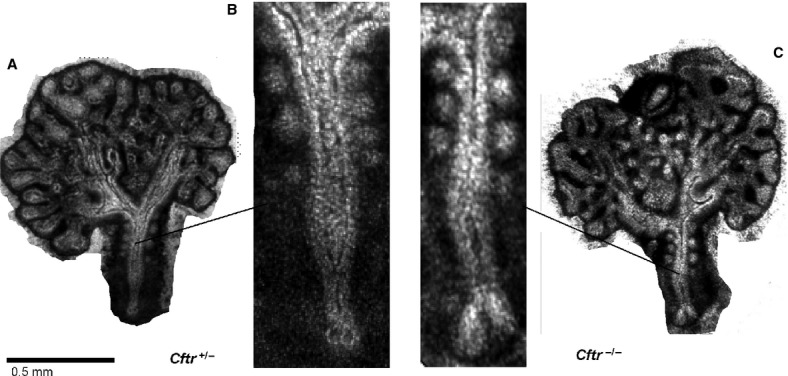
Embryonic lungs at 11.5 days gestation shown after 78 h in culture (A *cftr*^*+/−*^ and C *cftr*^*−/−*^). (B) Enlarged photographs of trachea from *cftr*^*+/−*^ (left) and *cftr*^*−/−*^ (right) embryos.

In order to determine whether the morphological differences observed in adult mouse tracheas occur early in development, lungs were dissected from mouse embryos and cultured for 72 h as described previously (Jesudason et al. 2000). There was no significant difference in trachea length, width above the bronchus (width 1) and below the larynx (width 2), and overall area between *cftr*^*+/−*^ and *cftr*^*−/−*^ mice at 11.5 days gestation after 6, 30, 54, and 78 h in culture (Table 1, Fig. 6).

**Table 1:** Morphometric data for cultured embryonic tracheas at different culture time points

Time in culture	Length (mm)	Width 1 (mm)	Width 2 (mm)	Area (mm^2^)
6	0.42 ± 0.03 (*cftr*^*+/−*^)	0.10 ± 0.005 (*cftr*^*+/−*^)	0.09 ± 0.005 (*cftr*^*+/−*^)	0.04 ± 0.003 (*cftr*^*+/−*^)
	0.43 ± 0.02 (*cftr*^*−/−*^)	0.09 ± 0.007 (*cftr*^*−/−*^)	0.1 ± 0.005 (*cftr*^*−/−*^)	0.04 ± 0.003 (*cftr*^*−/−*^)
30	0.45 ± 0.03 (*cftr*^*+/−*^)	0.12 ± 0.008 (*cftr*^*+/−*^)	0.11 ± 0.008 (*cftr*^*+/−*^)	0.11 ± 0.008 (*cftr*^*+/−*^)
	0.46 ± 0.02 (*cftr*^*−/−*^)	0.12 ± 0.006 (*cftr*^*−/−*^)	0.12 ± 0.009 (*cftr*^*−/−*^)	0.12 ± 0.009 (*cftr*^*−/−*^)
54	0.41 ± 0.03 (*cftr*^*+/−*^)	0.10 ± 0.006 (*cftr*^*+/−*^)	0.11 ± 0.01 (*cftr*^*+/−*^)	0.046 ± 0.005 (*cftr*^*+/−*^)
	0.43 ± 0.03 (*cftr*^*−/−*^)	0.11 ± 0.01 (*cftr*^*−/−*^)	0.10 ± 0.01 (*cftr*^*−/−*^)	0.06 ± 0.005 (*cftr*^*−/−*^)
78	0.43 ± 0.04 (*cftr*^*+/−*^)	0.11 ± 0.01 (*cftr*^*+/−*^)	0.11 ± 0.01 (*cftr*^*+/−*^)	0.04 ± 0.03 (*cftr*^*+/−*^)
	0.39 ± 0.06 (*cftr*^*−/−*^)	0.10 ± 0.01 (*cftr*^*−/−*^)	0.10 ± 0.02 (*cftr*^*−/−*^)	0.05 ± 0.03 (*cftr*^*−/−*^)

Tracheal measurements are shown for *cftr*^*+/−*^ (*n* = 10) and *cftr*^*−/−*^ (*n* = 10) embryos.

## Discussion

These data show a loss in the contractile capacity of the CF mouse trachea in response to CCh in Ca-free conditions. The reduction in contraction was agonist specific and occurred throughout the length of the trachea. Proximal cross-sectional areas of *cftr*^*−/−*^ tracheas and luminal areas were significantly smaller, but there was no difference in the area or distribution of smooth muscle. These observations indicate a defect in smooth muscle function in the absence of CFTR and raise some interesting questions regarding the mechanisms involved and the implications of a loss in contractile capacity in the human CF airway.

Tracheal smooth muscle contraction is primarily mediated by activation of muscarinic cholinergic receptors that couple to G_i/o_ and G_q_ receptors. M_3_/G_q_-coupled receptor signaling leads to activation of IP_3_ receptors and Ca release from the SR (Wray and Burdyga 2010). Cholinergic agonists can also initiate Ca influx by activation of a number of nonselective cation and receptor-operated channels, and store-operated Ca channels activated by SR depletion of Ca (Kajita and Yamaguchi 1993; Wang et al. 1997; Semenov et al. 2011). As the reduction in maximal response to CCh occurred in Ca-free conditions, this indicates a defect in the G_q_ receptor coupled pathway linked to Ca release from the SR. The response to CCh was also shorter in duration as indicated by the duration of the contractile response at 50% and *t*_1/2_. These data together with results from a study using a similar preparation (Bonvin et al. 2008) suggest decreases in sensitivity, strength, and duration of responsiveness to activation of the muscarinic receptor pathway.

Similar responses of *cftr*^*+/−*^ and *cftr*^*−/−*^ mice to caffeine and Ca release from the store via RyR's suggest that lack of CFTR is not reducing the capacity of the SR to release Ca as suggested previously (Michoud et al. 2009). We observed similar responses in *cftr*^*+/−*^ and *cftr*^*−/−*^ mice to activation from 5-HT. The cellular mechanism for 5-HT_2A_-induced contraction of ASM has been reported to involve an increase in intracellular Ca via the IP_3_ pathway similar to acetylcholine (Ach)-induced contraction (Yang et al. 1994). However, one study in the guinea pig trachea has shown that activation of 5-HT_2A_ does not involve phosphoinositide (PI) hydrolysis, but may involve Ca release from the SR via the RyR pathway (Watts et al. 1994). Contraction of equine tracheal myocytes can also occur via G_i_ receptor-coupled recruitment of the cyclic adenosine diphosphoribose (cADPR) signaling pathway and Ca release from the SR via RyR (Wang et al. 2004; Jude et al. 2008). This suggests that 5-HT may be activating an alternative signaling pathway to ACh activation, and the loss in contractility observed with CCh is due to downregulation of the pathway specific to ACh. This could be due to a reduction in M_3_ receptor expression and/or effects further downstream effecting IP_3_ activation and the resulting Ca signal. However, the relatively poor contraction obtained with 5-HT in this preparation may be insufficient to demonstrate any reduction in response in *cftr*^*−/−*^ mice. Therefore, a small contribution from nonspecific effects may add to our findings of a reduction in contractile response to CCh.

Results from the inhibitor H-1152 show that agonist binding has a minimal effect on rho kinase activation in tracheal smooth muscle of *cftr*^*+/−*^ or *cftr*^*−/−*^ mice. Although Rho kinase–mediated Ca sensitization has been shown to play a role in bronchial smooth muscle contraction (Chiba and Misawa 2004), Rho-kinase activity and Ca sensitivity has also been shown to vary between species (Shabir et al. 2004; Bai and Sanderson 2009). The airways of mice have shown substantially reduced Ca sensitivity compared to rat (Bai and Sanderson 2009). Our results suggest that in mouse trachea the reduction in contractility is therefore more likely to be due to a defective Ca-dependent pathway, although other Ca-independent pathways could be involved. Results also showed that the sustained component of the response to high- K^+^ in tracheal smooth muscle is ROK sensitive. An association between voltage-dependent Ca entry and ROK activation has also been reported in other smooth muscle types (Borysova et al. 2011). Rho kinase has been shown to play a key role in contraction by modulation of Ca influx through L-type Ca channels and through modulation of MLCP activity (Wang et al. 2004).The sustained component of the response to K^+^ depolarization in *cftr*^*−/−*^ is more ROK sensitive than in *cftr*^*+/−*^ mice; this result suggests that ROK activity is elevated in the CF airway. Interestingly, Rho A expression is reportedly increased in CFTR-deficient epithelial cells (Vandivier et al. 2009). This increase in ROK activity appears to maintain high-K^+^-induced contraction in *cftr*^*−*/*−*^ mice at normal levels, as in the absence of H-1152 there was no difference observed between groups. In the non-CF airway, contraction is thought to be primarily mediated by agonist-induced intracellular Ca release mechanisms rather than depolarization-mediated Ca influx (Sanderson et al. 2008). The significance of this finding therefore remains to be determined.

Morphological analysis showed that there was a reduction in the size of the trachea compared with controls as indicated by a reduction in the number of cartilage rings and the overall area of tracheal cross sections at the proximal end of the trachea. A recent study using *cftr*^tm1Unc^ mice reported proximal tracheal narrowing, but they did not observe a difference in the number of tracheal rings or epithelial abnormalities (Bonvin et al. 2008). These differences may be attributable to the variation in genetic background and type CF knockout mice, suggesting that the *cftr*^*tm1Cam*^ MF1/129 mutant possesses a more severe tracheal phenotype. We observed an increase in the area of epithelial layer lining the lumen which may contribute to the observed reduction in the lumen area. The decrease in lumen area as a percentage of cross-sectional area and decrease in circularity are consistent with observations in pigs and infants with CF (Meyerholz et al. 2010). This coincided with an increase in the area occupied by cartilage and connective tissue, suggesting the occurrence of remodeling. The reduction in luminal area does not appear to be due to a persistent hypercontractile state of ASM, as the amplitude of contraction in response to high-K^+^ depolarization and relaxation characteristics were similar to the control group. This is also strengthened by the similarities in ASM area and distribution between groups. We propose that the reduction in lumen area and circularity reflects the observed cartilage abnormalities (Bonvin et al. 2008).

Although a study by Pan et al. (2006) has shown a significant reduction in smooth muscle mass in the lower airways of CF mice, this does not appear to be the case in the upper airway as smooth muscle area and distribution in tracheal rings was similar between groups. This observation suggests that the reduction in ASM contractility in tracheal rings to CCh is due to functional abnormalities and not simply a reduction in the amount of ASM. The reduction in response is consistently demonstrated throughout the trachea, also indicating ASM functional changes. If the abnormalities in tracheal architecture (Bonvin et al. 2008 and our observations) were causing the reduction in contractility, you would expect to see a greater reduction in more proximal rings.

We have previously observed normal lung growth and peristalsis (Wallace et al. 2008) in embryonic lungs of *cftr*^*−/−*^ mice. We have also demonstrated in this study that there is no evidence of a reduction in tracheal caliber at this stage of development. This is consistent with a lack of upper tracheal constriction in new born *cftr*^*−/−*^ mice (Bonvin et al. 2008). These data suggest that CFTR has a less prominent role during development.

In conclusion, our results suggest a role for CFTR in maintaining ASM function. Consequences of upper tracheal stenosis and a reduction in smooth muscle tone in *cftr*^*−/−*^ mice may result in altered respiratory airflow as demonstrated by lower breathing rate in *cftr*^*−/−*^ mice (Bonvin et al. 2008). In the human CF airway, asthma-type symptoms of AHR may not be as apparent due to the underlying reduction in smooth muscle tone. These data would support the unexpected lack of evidence that bronchodilators are beneficial for treating CF airway obstruction and help to give us a better insight into CF airways disease.
